# Endovascular Management of Acute Lower Limb Ischemia Linked to Cocaine Abuse: A Case Report

**DOI:** 10.7759/cureus.58144

**Published:** 2024-04-12

**Authors:** Julián Andrés Muñoz Durán, Santiago Echeverri Isaza, Brayan Muñoz-Caicedo, José Miguel Hidalgo Oviedo

**Affiliations:** 1 Department of Radiology, Universidad de Antioquia, Medellín, COL; 2 Department of Interventional Radiology, Hospital Pablo Tobón Uribe, Medellín, COL

**Keywords:** endovascular procedures, nitroglycerin, lower limbs, ischemia, acute effects, cocaine abuse

## Abstract

Cocaine abuse is a public health concern with well-documented cardiovascular complications. However, acute limb ischemia remains a rare and underreported consequence. We present a case of a 36-year-old man with acute right lower limb ischemia following heavy cocaine use, successfully managed with systemic heparin and intra-arterial nitroglycerin. The case highlights considering cocaine as a potential cause of acute limb ischemia and the efficacy of endovascular therapy. Further case reports with this diagnosis and their management are crucial for establishing the best strategies and improving outcomes in these scenarios.

## Introduction

Cocaine is a highly addictive illicit drug considered a public health problem. It has many common cardiovascular and cerebrovascular effects, such as myocardial infarction, arrhythmias, stroke, aortic dissection, thrombosis, and embolism. Acute lower limb ischemia is a rare and probably underreported complication, with only five studies about this topic in the literature, all case reports, which included eight patients until August 2023 [1-3].

The medical significance of acute limb ischemia is that it requires urgent attention and treatment. Although it is unclear what causes the condition, some theories suggest that it may be related to local vasoconstriction, thrombosis, or emboli formation. These are usually young patients recently exposed to heavy cocaine consumption, followed by arterial insufficiency symptoms and signs. In imaging studies, the main findings could be arterial spasms or thrombosis, which lead to management options like vasodilators or heparin. Diagnosis can be challenging because signs and symptoms often mimic other conditions.

We present a case of an adult male patient with acute right lower limb ischemia related to recent cocaine abuse with successful endovascular management.

## Case presentation

**Figure 1:**
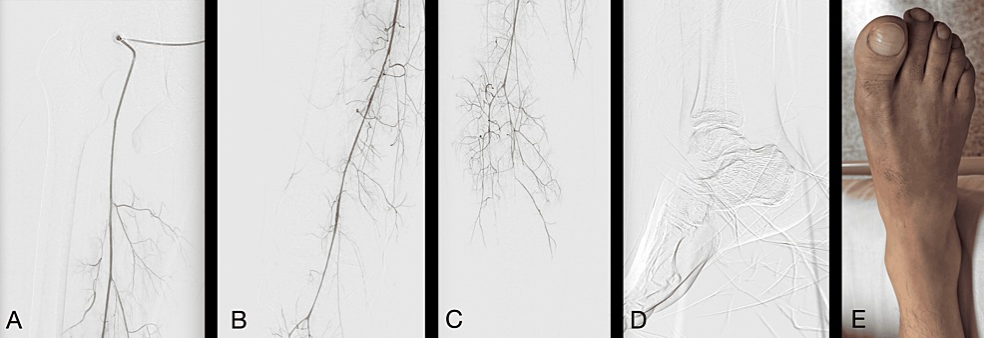
The patient’s initial right lower limb arteriography and clinical photo of his right foot at hospital admission (Philips Azurion 7). (A) Decreased caliber of the superficial femoral artery. (B) Spasm in the popliteal artery. (C) The tibiofibular trunk and the anterior tibial artery exhibit a decreased diameter, with filling visible only up to the proximal third of the leg. (D) Absence of contrast in the arteries of the middle and distal thirds of the leg and foot. (E) Photograph of the patient's right foot taken upon admission. Photo with the patient’s consent.

A previously asymptomatic 36-year-old man with a history of massive cocaine inhalation over the past week presented to the emergency department with pain in his right lower limb and progressive inability to walk, culminating in complete rest for the past two days. Physical examination revealed coldness and bluish discoloration in the distal part of the right lower limb, absent capillary refill, and pain during active leg movement. No pedal or tibial pulses were palpable on the right side. With the clinical diagnosis of acute ischemia, he underwent arteriography, which showed severe and diffuse arterial vasospasm of the right lower limb (Figure 1).

**Figure 2:**
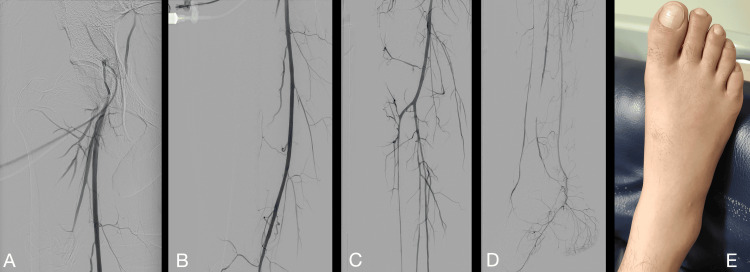
Follow-up right lower limb arteriography and clinical image of the right foot (Philips Azurion 7). (A) Recovery of the usual caliber of the superficial femoral artery. (B) Absence of spasm in the popliteal artery. (C) The tibiofibular trunk and anterior tibial artery appear at their usual diameter, with visible filling along the entire leg length. (D) Depicts contrast medium presence in the distal arteries of the leg and foot. (E) Post-treatment photograph of the patient's foot. Photo with patient’s consent.

With the diagnosis of diffuse right lower limb arterial supra popliteal and infrapopliteal spasms, the patient was managed with nitroglycerine, using a total intraarterial dose of 1.000 micrograms (µg) with posterior intraarterial infusion (1 µg/Kg/min). The patient was also managed with systemic intravenous heparin, maintaining an activated partial thromboplastin time between 35 and 45 seconds and continuous monitoring. Twenty-four hours later, the patient exhibited complete symptomatic improvement and a remarkable clinical improvement with normal skin color, capillary refill, and pedal pulses. A control angiography was made (Figure 2).

The patient also had rhabdomyolysis but without kidney failure. After laboratory and clinical analysis, a diagnosis of right lower limb ischemia explained by cocaine intoxication was made. The patient received a toxicologic evaluation and platelet antiaggregation prescription and was discharged with follow-up recommendations. Unfortunately, he was lost from institutional monitoring.

## Discussion

Cocaine consumption represents a public health concern and varies globally, being more common in high-income countries. The reported global prevalence is 0.4% in individuals aged 15-64 years, ranging from 0.95% in South America up to 1.9% in North America [4]. Notably, intoxication with this drug emerges as the leading cause of acute drug-related emergency department consultations, accounting for 162 visits per 100,000 population in the United States [5,6].

It is a natural alkaloid extracted from the leaves of the coca plant (*Erythroxylum coca*) through a step series. It has three primary metabolites: benzoylecgonine, ecgonine methyl ester, and norcocaine [3,7]. Other metabolites result from practices, such as drug heating or concomitant consumption with other substances, like ethanol (i.e., cocaethylene) [8]. Special mention requires adulterants added to cocaine, for example, levamisole, clenbuterol, or arsenic, interpreting the cocaine net effects in the vascular beds tough to isolate [1,9].

The drug's kinetics varies with the administration route (intravenous, subcutaneous, intramuscular, smoked, or mucosal-oral, sublingual, snorted, rectal, or vaginal) [3,5,7,10-12]. For the mucosal route, the onset typically occurs within five to 25 minutes, the peak effect between 30 and 60 minutes, the action duration of 60 to 180 minutes, and a half-life spanning 60 to 120 minutes [10].

Cocaine's vasoconstrictive effects are well-established, mediated by direct receptor stimulation and indirect mechanisms, including impaired nitric oxide production, altered intracellular calcium handling, elevated levels of endothelin-1, and endothelial dysfunction. Moreover, cocaine's prothrombotic effects, stemming from endothelial damage, increased fibrinogen levels, enhanced von Willebrand factor activity, elevated plasminogen activator inhibitor activity, and augmented platelet aggregation, contribute to thrombotic and embolic multisystemic complications [2,5,10-14]. Understanding this physiopathology supports management strategies, like the use of heparin and vasodilators.

Case reports in the literature highlight demographic patterns in lower limb ischemia secondary to cocaine, primarily affecting males (62%) with an average age of 38 years. The condition is associated with recent cocaine use. Clinical presentation includes localized pain, claudication of variable duration (weeks), acute lower limb arterial insufficiency with coldness, diminished or absent pulses, delayed capillary refill, and even tissue necrosis in advanced cases. The physical examination with signs of poor arterial circulation as pallor or bluish discoloration of the skin, cold to palpation, diminished or absent pulses, retarded capillary refill, and even necrosis areas, mainly in patients with severe vascular compromise and non-respond to initial management. Diagnostic findings include arterial vasospasms, occlusion, or thrombosis (62.5%) in vascular territories that can be localized from the external iliac artery or downstream. Rhabdomyolysis and thrombosis may be present [1,15].

The management is variable, including systemic heparin, systemic vasodilators (iloprost, nitroglycerine), intraarterial therapy (iloprost, nitroglycerine, guanethidine, and ropivacaine), balloon angioplasty, thrombolysis (urokinase), and surgical thrombectomy. Although heparin remains the most frequent intervention, its administration may pose risks, such as central nervous system bleeding that happened in one case report. Most patients improved with these strategies, mainly surgical thrombectomy; however, the data are limited for recommendations [1,2,5,16].

Finally, this is the ninth reported patient, to the best of our knowledge, with lower limb ischemia diagnosed secondary to cocaine. This patient was successfully managed with endovascular therapy, a valuable experience in the literature. While the intrinsic limitations of a case report and the lost follow-up hinder its generalizability, continued publication of this diagnosis and interventions is crucial for refining the best and least invasive management strategies.

## Conclusions

In a young patient with isolated acute arterial limb insufficiency or ischemia, it is essential to consider cocaine-associated arterial vasoconstriction or coagulation as highly probable etiologies in the differential diagnosis. In our case, arteriography confirmed the extensive and diffuse vasoconstriction from the femoral common artery and downstream but was more severe in the leg and foot. The successful management of systemic heparin and intra-arterial nitroglycerin is a valuable experience for a case report aiming to share the successful endovascular treatment.
